# Ca^2+^ regulation of glutamate release from inner hair cells of hearing mice

**DOI:** 10.1073/pnas.2311539120

**Published:** 2023-11-29

**Authors:** Lina María Jaime Tobón, Tobias Moser

**Affiliations:** ^a^Auditory Neuroscience and Synaptic Nanophysiology Group, Max Planck Institute for Multidisciplinary Sciences, Göttingen 37077, Germany; ^b^Institute for Auditory Neuroscience, University Medical Center Göttingen, Göttingen 37075, Germany; ^c^Collaborative Research Center 889, University of Göttingen, Göttingen 37075, Germany; ^d^Multiscale Bioimaging of Excitable Cells, Cluster of Excellence, Göttingen 37075, Germany

**Keywords:** cochlea, sound encoding, active zone, calcium channel, paired recordings

## Abstract

In our hearing organ, sound is encoded at ribbon synapses formed by inner hair cells (IHCs) and spiral ganglion neurons (SGNs). How the underlying synaptic vesicle (SV) release is controlled by Ca^2+^ in IHCs of hearing animals remained to be investigated. Here, we performed patch-clamp SGN recordings of the initial rate of release evoked by brief IHC Ca^2+^-influx in an ex vivo cochlear preparation from hearing mice. We aimed to closely mimic physiological conditions by perforated-patch recordings from IHCs kept at the physiological resting potential and at body temperature. We found release to relate supralinearly to Ca^2+^-influx (power, *m*: 4.3) when manipulating the [Ca^2+^] available for SV release by Zn^2+^-flicker-blocking of the single Ca^2+^-channel current. In contrast, a near linear Ca^2+^ dependence (*m*: 1.2 to 1.5) was observed when varying the number of open Ca^2+^-channels during deactivating Ca^2+^-currents and by dihydropyridine channel-inhibition. Concurrent changes of number and current of open Ca^2+^-channels over the range of physiological depolarizations revealed *m*: 1.8. These findings indicate that SV release requires ~4 Ca^2+^-ions to bind to their Ca^2+^-sensor of fusion. We interpret the near linear Ca^2+^-dependence of release during manipulations that change the number of open Ca^2+^-channels to reflect control of SV release by the high [Ca^2+^] in the Ca^2+^-nanodomain of one or few nearby Ca^2+^-channels. We propose that a combination of Ca^2+^ nanodomain control and supralinear intrinsic Ca^2+^-dependence of fusion optimally links SV release to the timing and amplitude of the IHC receptor potential and separates it from other IHC Ca^2+^-signals unrelated to afferent synaptic transmission.

## Significance

The first synapse of the auditory pathway faithfully encodes time and intensity of sounds. Ca^2+^ influx into the inner hair cell via voltage-gated Ca^2+^ channels links the receptor potential to synaptic vesicle (SV) release. Understanding this Ca^2+^ signaling and the Ca^2+^ dependence of SV release is fundamental for deciphering sound encoding. Pre- and postsynaptic patch-clamp recordings in cochleae of hearing mice revealed a supralinear dependence of release on [Ca^2+^] at the SV release site. Yet, release reports the receptor potential in a near-linear manner. This indicates that [Ca^2+^] at the SV release site is governed by one or few nearby Ca^2+^ channels. The supralinear Ca^2+^ dependence of SV release likely reflects the properties of the Ca^2+^ sensor of SV release.

The sense of hearing relies on precise and tireless encoding of sounds (1, 2). The inner hair cell (IHC) receptor potential represents the broad range of audible sound pressures (or intensity) (3). Each IHC forms ribbon synapses with several type I spiral ganglion neurons (SGNs) that relay the auditory information to the brainstem. Most SGNs receive input from a single IHC active zone (AZ) (4, 5). Their spontaneous (up to 150 spikes per second) and sound-evoked (up to several hundreds of spikes per second) firing rates (6–10) place high demands on the rate of synaptic vesicle (SV) release and on the efficiency of synaptic transmission. Indeed, vivid fusion of roughly a dozen readily releasable SVs accommodates initial rates of exocytosis of >1,000 SVs per second at a single AZ and sustained exocytosis of hundreds of SVs per second (e.g., refs. 11 and 12). The rate of exocytosis reflects the IHC potential (12–16) enabling sound intensity to be encoded as the rate of glutamate release and consequent SGN firing (6–10). The large postsynaptic cluster of ionotropic glutamate receptors enables big excitatory postsynaptic currents (EPSCs) (17) whereby release of an individual SV can efficiently elicit an action potential in the compact postsynaptic element of the SGN (18–20). Endowing each SV release event with such significance for information processing requires the release to be tightly controlled by voltage-gated presynaptic Ca^2+^ influx at rest and during receptor potentials. Avoiding impact on release of non-AZ Ca^2+^ signals, e.g., arising from mechanoelectrical transduction (e.g., ref. 21), efferent transmission (e.g., ref. 22), and Ca^2+^ release from internal stores (e.g. ref. 23) seems critical in this respect.

The control of SV release builds on three key elements: i) voltage-gated Ca^2+^ channels, ii) the localization of Ca^2+^ channel(s) with respect to SV release sites and cytosolic Ca^2+^ buffering, as well as iii) the Ca^2+^ sensor of the SV. IHC AZs rely on L-type Ca_V_1.3 Ca^2+^ channels (24–26) that activate at low voltages (27, 28) and inactivate very little (11, 24, 28, 29). IHC AZs employ multidomain proteins, such as bassoon (30, 31), rab interacting molecule 2 (32), and RIM-binding protein 2 (33), to cluster 20 to 300 Ca_V_1.3 channels as a function of AZ size (34) underneath the presynaptic density (35) at the base of the ribbon (13, 35). While the morphological identity of the readily releasable pool of SVs (RRP) needs further investigation (36), recent electron tomography studies of stimulated AZs indicate that membrane proximal SVs, tethered or docked to the AZ, comprise the structural correlate of the RRP (32, 37, 38). The spatial coupling of Ca^2+^ channels and SV release sites at IHC AZ has been studied by experiments and modeling in the past (12–14, 35, 39, 40). A Ca^2+^ nanodomain of one or few Ca^2+^ channels has been suggested to control SV release sites at the majority of IHC AZs after the onset of hearing. Yet, uncertainties remained due to methodological shortcomings of membrane capacitance (C_m_) measurements and imaging of glutamate release (using the intensity-based glutamate-sensing fluorescent reporter iGluSNfR) (13, 14, 35, 39, 40). Both of these techniques, employed in past work, lack the sensitivity to resolve the initial rate of release from a full RRP of SVs, which is required for faithful analysis of the Ca^2+^ dependence of fusion. Hence, depolarizations were often ≥20 ms long, depleting not only the RRP but also releasing newly replenished SVs (35, 39). Moreover, whole-cell C_m_ measurements report Ca^2+^-triggered membrane fusion not limited to SV exocytosis (41) and sum over all IHC synapses, that vary in properties (14, 35, 42–44).

Paired pre- and postsynaptic patch-clamp recordings e.g. refs. 12, 15, 16, and 45 offer the specificity, sensitivity, and temporal resolution to study initial SV release at individual AZs. However, to our knowledge, a characterization of the Ca^2+^ dependence SV release in IHCs from hearing animals using this technique had yet to be performed. Moreover, the search for the IHC Ca^2+^ sensor of exocytosis is ongoing. The best candidate is otoferlin (46–48), a multi-C_2_ domain hair cell–specific protein that is disrupted in human genetic deafness DFNB9 (49), an auditory synaptopathy (review in refs. 50 and 51). Cooperative binding of 4 to 5 Ca^2+^ ions seems required for IHC exocytosis according to C_m_ measurements upon Ca^2+^ uncaging (52). This finding seems compatible with Ca^2+^ binding to otoferlin’s C_2_ domains (46, 47). Here, we investigated the Ca^2+^ dependence of physiological IHC SV release and its coupling to Ca_V_1.3 Ca^2+^ channels using simultaneous pre- and postsynaptic patch-clamp recordings from IHCs and SGNs of hearing mice. We mimicked physiological conditions by perforated-patch recordings from IHCs that we kept at the physiological resting potential and at body temperature. The results indicate that the average IHC SV release requires binding of ~4 Ca^2+^ ions from one or few neighboring Ca^2+^ channels.

## Results and Discussion

### Estimating the Intrinsic Ca^2+^ Dependence of SV Release.

**Fig. 1.**
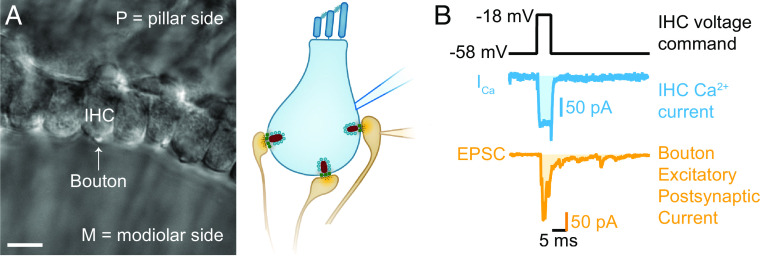
Paired IHC-bouton patch-clamp recordings to study the biophysical properties of individual IHC ribbon synapses. (*A*) Differential interference contrast (DIC) image of an explanted mouse organ of Corti. In this example, supporting cells from the modiolar side (M) were removed to gain access to the IHCs and their contacting boutons. (*B*) Evoked release was recorded using depolarizing pulses (black trace), triggering whole cell IHC Ca^2+^ influx (I_Ca_, blue trace), and concomitant release of neurotransmitter (detected as postsynaptic EPSC, orange trace). Ca^2+^ charge and EPSC charge were estimated by taking the integrals of the currents (shaded light blue and light orange areas).

How many Ca^2+^ ions have to bind to the Ca^2+^ sensor of a fusion competent IHC SV for it to release? We addressed this question using apical cochlear coils, freshly dissected from hearing mice (c57BL/6N mice between postnatal day 14 to 23). Aiming to match physiological conditions as closely as possible in our ex vivo preparation (Fig. 1), we used the perforated-patch configuration to stimulate the IHCs by voltage-clamp depolarizations from −58 mV, which is near their putative resting potential (53), at near physiological temperature (at 32 to 37 °C) and an extracellular solution with [Ca^2+^]_e_ of 1.3 mM mimicking the perilymph bathing the IHC synapses in vivo (54). Perforated-patch recordings provide the least alteration of the cytosolic composition and metabolic state and enable long-lasting recordings from IHCs with low rundown of voltage gated Ca^2+^ current and exocytosis (*SI Appendix*, Fig. S1 and refs. 11, 55, and 56). The synaptic release of neurotransmitter was measured by ruptured-patch clamp recordings of the postsynaptic bouton from one of the connecting SGNs on either the pillar or the modiolar (Fig. 1*A*) side of the IHC.

**Fig. 2.**
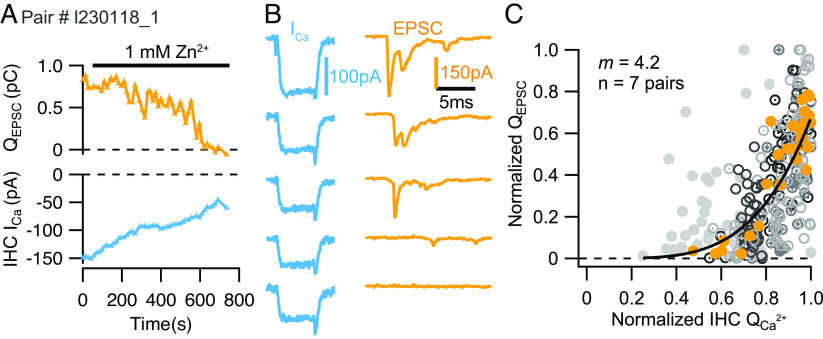
Estimating the intrinsic Ca^2+^ dependence of SV release. (*A* and *B*) Slow perfusion of 1 mM Zn^2+^ to reduce the effective fusogenic Ca^2+^ signal (reflected in a decrease of the whole-cell current, blue) and the concomitant neurotransmitter release (Q_EPSC_; orange) evoked by 5 ms step depolarizations. (*C*) Scatter plot of the normalized elicited EPSC charges (Q_EPSC_) vs. the corresponding normalized Ca^2+^ current integrals (Q_Ca_): different markers and shades of gray for the different pairs and orange for the exemplary pair shown in *A* and *B*. The solid line is a least-squares fit of a power function [Q_EPSC_ = a(Q_Ca_)*^m^*] to the pooled normalized data, which revealed a supralinear relationship of neurotransmitter release (*m_Zn_* = 4.2; n = 7 pairs).

We employed brief (5 ms) depolarizations to the potential eliciting maximal Ca^2+^ influx (−19 mV) to determine initial release as the integrated EPSC (Q_EPSC_) without risking impact of RRP depletion and rundown of exocytosis (*SI Appendix*, Fig. S1). In the first type of experiment, we slowly reduced Ca^2+^ influx by perfusing the preparation with extracellular solution containing 1 mM Zn^2+^ (Fig. 2 *A* and *B*). Zn^2+^ causes a rapid (microsecond scale) flicker block of L-type Ca^2+^ channels but does not alter their open probability (57). We argue that this leads to a reduction of the *effective* Ca^2+^ signal that is relevant for its fusogenic action, given the limited speed of Ca^2+^ triggered fusion in IHCs (minimal time to peak release ~2 ms) (52).

We related changes of release at individual synapses (ΔQ_EPSC_) to the change of the integrated IHC Ca^2+^ influx (IHC ΔQ_Ca_, a proxy for the change of the average synaptic Ca^2+^ influx). While not knowing the [Ca^2+^] at the SV release site, this approach assesses initial release at different *effective* Ca^2+^ signals to approximate the Ca^2+^ binding to the Ca^2+^ sensor of fusion (intrinsic Ca^2+^ dependence of SV release). We fitted power functions [Q_EPSC_ = a(Q_Ca_)*^m^*] to the observed supralinear relationships for individual synapses (*SI Appendix*, Fig. S2) and found an average power *m_Zn_* of 4.3 ± 0.6 (SEM, SD: 1.6, n = 7). The fit to normalized pooled data also yielded an *m_Zn_* of 4.2 (Fig. 2*C*). We propose this to reflect the supralinear intrinsic Ca^2+^ dependence of SV release that results from the need for ~4 Ca^2+^ ions to bind to the Ca^2+^ sensor to trigger SV fusion. We note that changing the single channel current by manipulating [Ca^2+^]_e_ would be a more direct approach to determine the intrinsic Ca^2+^ dependence of SV release. But, in our hands, this approach was less practical for the challenging paired recordings as it risks the IHC stability at µM concentrations of [Ca^2+^]_e_. Previous C_m_ recordings modulated IHC exocytosis by changes in [Ca^2+^]_e_ and obtained an *m* value of 2.94 ± 0.70 (35), which might reflect the above mentioned risk of underestimation when using RRP depleting stimuli. Alternatively, the lower value may point to differences between the two approaches for changing the fusogenic Ca^2+^ signal.

### Investigating the Coupling of Ca^2+^ Channel and SV Release in IHCs of Hearing Mice.

Using the above estimated intrinsic Ca^2+^ dependence of SV release as a reference, we next studied the apparent Ca^2+^ dependence of SV release while manipulating the number of open Ca^2+^ channels. This classical approach has been applied to various synaptic preparations to estimate how Ca^2+^ channels control SV fusion (13, 58–61). Estimates of *m* lower than that of the intrinsic Ca^2+^ dependence of SV release indicate dominance of the Ca^2+^ nanodomain contributed by a single Ca^2+^ channel located within few nanometers from the SV release site. The argument is that a fusion-competent SV will be released unconditionally if a channel at such close distance opens because the ensuing high [Ca^2+^] of ≥100 µM (62) experienced by the vesicular Ca^2+^ sensor will drive SV fusion. Then, each opening will lead to a release, such that SV release increases linearly with the number of release site-coupled open Ca^2+^ channels. This condition is referred to as Ca^2+^ nanodomain control of exocytosis and implies a value *m* = 1 (58, 63). We note that the potential caveat of heterogeneous Ca^2+^ channel activation across synapses (14, 43, 44) affecting the *m* estimation when relating single synapse release to the whole IHC Ca^2+^ influx (64) seems less relevant if analysis is performed at a fixed potential that activates most if not all Ca^2+^ channels.

**Fig. 3.**
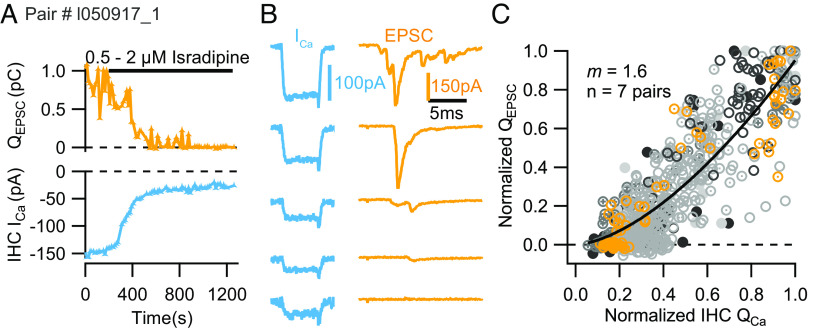
Apparent Ca^2+^ dependence of SV release during dihydropyridine-mediated reduction of the number of open Ca^2+^ channels in IHCs. (*A* and *B*) Slow perfusion of 0.5 to 2 µM of the dihydropyridine isradipine progressively reduced the IHC Ca^2+^ current integrals (Q_Ca_) and the elicited EPSC charge (Q_EPSC_) in an exemplary paired recording. (*C*) Scatter plot of the normalized elicited EPSC charges (Q_EPSC_) vs. the corresponding normalized Ca^2+^ current integrals (Q_Ca_): different markers and shades of gray for the different pairs and orange for the exemplary pair shown in *A* and *B*. Fitting a power function to the normalized population data for Q_EPSC_ and Q_Ca_ revealed a *m_isradipine_* estimate of 1.6 (n = 7 pairs).

In the first approach, we slowly perfused the dihydropyridine L-type Ca^2+^ channel blocker isradipine (at concentrations between 0.5 to 2 µM). Different from the Zn^2+^-flicker-block of the channel pore, binding of dihydropyridines to L-type Ca^2+^ channels shifts them to the long-lasting nonconducting “mode zero” but does not affect the single-channel current amplitude (65). Responses to depolarizations of 5 ms to −19 mV showed the expected reduction of Ca^2+^ influx and ensuing decline of Q_EPSC_ (Fig. 3 *A* and *B*).

**Fig. 4.**
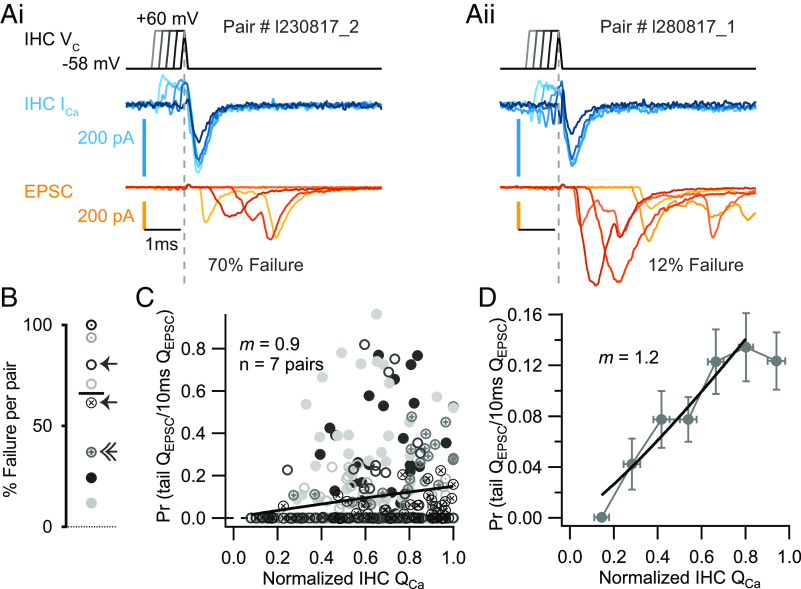
Apparent Ca^2+^ dependence of SV release during variation of the number of open Ca^2+^ channels in deactivating Ca^2+^ tail-currents in IHCs. (*A*, *i* and *ii*) Presynaptic voltage steps of increasing duration (0 to 2 ms) from −58 mV to +60 mV were used to titrate the number of open Ca^2+^ channels that contribute Ca^2+^ influx during the deactivating (tail) current upon repolarization to −58 mV. Increasing the length of the predepolarization increased the amplitude of the Ca^2+^ tail current (blue traces) and the size of the postsynaptic response (orange traces). (*B*) Failures of tail currents to evoke an EPSC were prominent and varied from pair to pair: Some pairs had a high percentage of failures (example in *A*, *i*), while others had a low percentage of failures (example in *A*, *ii*). Failures in synaptic transmission persisted even with a higher extracellular [Ca^2+^] (arrow: 2 mM; double arrow: 3 mM). Different markers and shades of gray for the different pairs (n = 8). Black bar corresponds to the median. (*C*) The evoked Q_EPSC_ was normalized to the responses elicited by 10 ms voltage steps to −19 mV that fully release the RRP to obtain the release probability (P_r_: tail Q_EPSC_/10 ms Q_EPSC_) and plotted vs. the normalized presynaptic Q_Ca_: different markers and shades of gray for the different pairs (n = 7). The solid line is a least-squares fit of a power function [Q_EPSC_ = a(Q_Ca_)*^m^*] to the population data yielding an *m_tails_* of 0.9 (n = 7 pairs). (*D*) Power function fit to the binned data (bin size ~ 0.15; data points are mean ± SEM) from *B* resulted in *m_tails_* of 1.2.

However, contrary to Zn^2+^ block, Q_EPSC_ decreased more gradually and persisted even for small Q_Ca_ values, indicating a near linear apparent Ca^2+^ dependence. By fitting the power function to the Q_EPSC_–Q_Ca_ relationships obtained for the individual recordings, we estimated an *m_isradipine_* of 1.5 ± 0.1 (SEM, SD: 0.2, n = 7; *SI Appendix*, Fig. S3) which is significantly lower than the *m_Zn_* (4.3, *P* = 0.0098). The fit to the pooled and normalized data returned a similar *m_isradipine_* of 1.6 (Fig. 3*C*). For a second approach, we aimed to titrate the number of open Ca^2+^ channels contributing to Ca^2+^ influx during brief deactivating (“tail”) currents after depolarizations to +60 mV of varying durations (0 to 2 ms). Depolarization near or beyond the reversal potential for Ca^2+^ ions does not permit Ca^2+^ influx. Repolarization (to −58 mV) causes Ca^2+^ tail currents whose amplitudes depend on the number of channels which have opened during the +60 mV depolarization. Thus, increasing the duration of the predepolarization recruits more open Ca^2+^ channels and increases the amplitude of the tail current (Fig. 4 *A*, *i* and *ii*, *Upper* and *Middle*), as shown in other synaptic preparations (61, 66, 67). To minimize the impact of capacitive currents, we ramped the voltage up and down at 1,180 mV/ms in addition to applying P/n correction. With this type of stimulation, synaptic transmission had a considerable number of failures (Fig. 4 *A*, *i* and *ii* and Fig. 4*B* and *SI Appendix*, Fig. S4 *A*–*C*), reaching 100% for one pair. This pair was excluded from the next steps. The percentage of failures of transmission did not decrease with higher extracellular [Ca^2+^] (Fig. 4*B*; arrow: 2 mM; double arrow: 3 mM).

For analysis, we normalized the tail Q_EPSC_ to the responses elicited by 10 ms voltage steps to −19 mV that we expect to fully release the RRP (35). The resulting release probability (P_r_) was plotted against the normalized IHC Q_Ca_ (Fig. 4*C*) and the relationship was described by a power function with *m_tail_* of 0.9 (fit to the combined data of 7 pairs). The power fit to the binned data (bin size ~ 0.15, Fig. 4*D*) yielded a power *m_tail_* of 1.2. Power fits to the normalized and binned normalized data gave *m_tail_* of 1.4 and 1.3, respectively (*SI Appendix*, Fig. S4 *D* and *E*). The estimates of *m* from both manipulations of the number of open Ca^2+^ channels are lower than the *m* estimate obtained for the intrinsic Ca^2+^ dependence with Zn^2+^ (*m_Zn_* = 4.3). We conclude from the lower apparent Ca^2+^ dependence that the Ca^2+^ at the SV release site is dominated by one or few Ca^2+^ channels, with limited overlap of their Ca^2+^ domains (63).

### Estimating the Apparent Ca^2+^ Dependence of SV Release during Physiological IHC Depolarization.

**Fig. 5.**
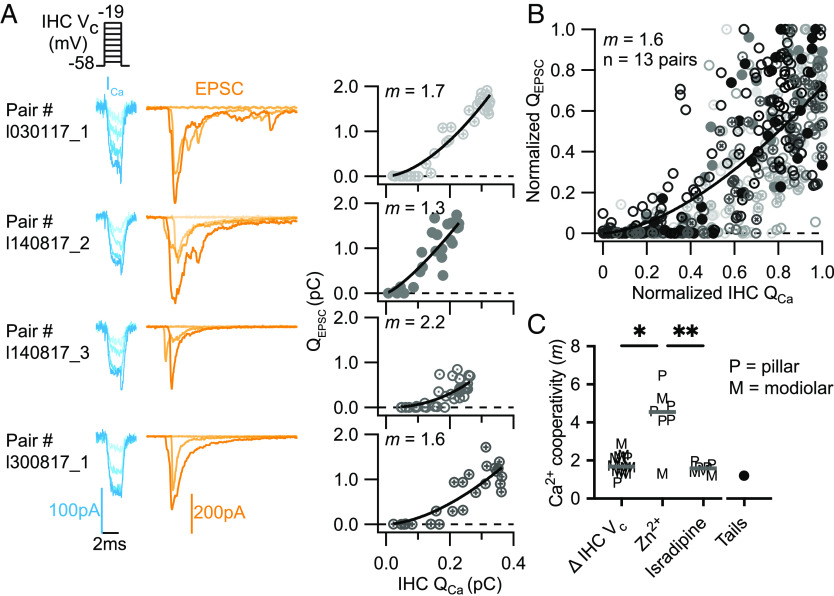
Few nearby Ca^2+^ channels control release in the range of IHC receptor potentials. (*A*) The IHC was depolarized for 2 ms to different potentials ranging from −57 to −19 mV, triggering different presynaptic Ca^2+^ currents (blue traces) and the ensuing release that elicited postsynaptic currents (orange traces). Shown here are representative traces from 4 different synapses. (*Right* column) Plotting EPSC charge (Q_EPSC_) vs. the IHC Ca^2+^ current integrals (Q_Ca_) for the corresponding data shown in the *Left* column reveals Ca^2+^ cooperativities (*m*_Δ__V_**) ranging from 1.3 to 2.2. (*B*) Scatter plot of normalized Q_EPSC_ vs. the corresponding normalized Q_Ca_: different markers and shades of gray for the different pairs. The solid line is a least-squares fit of a power function [Q_EPSC_ = a(Q_Ca_)*^m^*] to the data yielding a Ca^2+^ cooperativity (*m*) of 1.6 (n = 13 pairs). (*C*) Ca^2+^ cooperativities estimated for the different manipulations. Predominant changes in the number of open Ca^2+^ channels yielded significantly lower Ca^2+^ cooperativities than changes in the single Ca^2+^ channel current. Brown–Forsythe (*P* = 0.0015) and Welch ANOVA (*P* = 0.0022) tests followed by a Dunnett’s T3 multiple comparisons test (*m*_Δ__V_** vs *m_isradipine_*: n.s., *P* = 0.5529; *m*_*ΔV*_ vs *m_Zn_*: *P = 0.0110; *m_isradipine_* vs *m_Zn_*: **P = 0.0098). *m_tails_* is shown as a comparison but was not included in the statistical tests. P indicates the boutons contacting the pillar side of the IHC; M indicates the boutons contacting the modiolar side of the IHC. Gray bar corresponds to the median.

Finally, we addressed Ca^2+^ channel-SV release coupling during IHC depolarizations in the range of physiological receptor potentials (3). We employed very short (2 ms) depolarizing pulses to different potentials ranging from −57 mV to −19 mV in randomized steps with a resolution of 2 mV (Fig. 5*A*). This protocol varies Ca^2+^ influx via changing the channel open probability as well as the single channel current by changing the driving force for Ca^2+^. The short pulse duration was aimed to assess initial release avoiding effects of RRP depletion. We encountered failures of synaptic transmission, although to a lesser extent than for the tail current experiments (compare *SI Appendix*, Figs. S4 and S5). As illustrated for 4 exemplary recordings (Fig. 5*A*), we typically found a low power *m*_Δ__V_** of the Q_EPSC_ vs. Q_Ca_ relationships. On average, *m*_Δ__V_** was 1.8 ± 0.15 (SEM, SD: 0.5, n = 13 pairs) which is significantly lower than the *m_Zn_* upon manipulating the single Ca^2+^ channel current by Zn^2+^ (*m_Zn_* = 4.3, *P* = 0.0110, Fig. 5*C*). The fit to the normalized pooled data revealed an *m_ΔV_* of 1.6 (Fig. 5*B*). We take this data to suggest that a Ca^2+^ nanodomain-like coupling of channels and SV release sites controls SV release during brief stimuli or physiological receptor potentials in response to low-frequency acoustic stimulation.

We refrained from extending the Q_EPSC_ vs. Q_Ca_ relationships to very depolarized potentials that maximize open probability but reduce the driving force for Ca^2+^ (ref. 12) because i) the focus here was on Ca^2+^ channel-SV release coupling during IHC depolarizations in the range of physiological receptor potentials and ii) we were concerned with the faithful isolation of I_Ca_ from currents, e.g., mediated by Cs^+^ at these depolarized potentials. In summary, we obtained lower *m* estimates when primarily changing Ca^2+^ influx by varying the number of open channels (*m_isradipine_*, *m_tail_*, and *m_ΔV_*) than when changing the apparent single channel current (*m_Zn_*, Fig. 5*C*). This difference indicates a Ca^2+^ nanodomain control of release in the range of physiological receptor potentials.

## Discussion

Two decades after the first membrane capacitance recordings of Ca^2+^-triggered exocytosis of IHCs (11), the present paired pre- and postsynaptic recordings address the longstanding question of the physiological Ca^2+^ dependence of SV release at individual afferent synapses of mammalian IHCs after hearing onset. Our challenges when applying paired recordings from hair cell afferent synapses, that were pioneered for frog auditory papilla (15) and the organ of Corti of pre-hearing rats (12), were manyfold. They included i) the low success rate of simultaneous patch-clamp recording from tiny SGNs boutons and IHCs, ii) the ex vivo recording conditions aimed at physiological IHC condition, temperature and [Ca^2+^]_e_, iii) the stochastic, variably sized and shaped release events of single AZs, as well as the iv) need to sustain the recordings, Ca^2+^ influx and SV release for the time required for pharmacological manipulation of Ca^2+^ influx (56). Yet, this effort is justified by the sensitivity, kinetics and specificity of these experiments that enabled analysis of how Ca^2+^ influx controls SV release at single AZs unaffected by RRP depletion and in isolation from other Ca^2+^ dependent processes such as SV endocytosis and SV replenishment. Considering the present results and previous studies, we conclude that SV fusion at IHC synapses of hearing mice combines supralinear intrinsic Ca^2+^ dependence of the Ca^2+^ sensor with control by few Ca_V_1.3 Ca^2+^ channels with ≤15 nm effective coupling distance to the SV release site.

The observed ~4th power Ca^2+^ dependence of initial SV release (m*_Zn_*: 4.3) is consistent with estimates at other synapses such as neuromuscular junction (68), retinal bipolar neurons (69) and calyx of Held (70). However, the intrinsic Ca^2+^ dependence of exocytosis seemed to be less clear for hair cell synapses. A previous study combining Ca^2+^ uncaging and C_m_ recordings in IHCs (52) estimated a 4th to 5th power Ca^2+^ dependence of Ca^2+^ triggered membrane fusion. There, step-like increments in the global cytosolic [Ca^2+^] beyond 10 µM elicited C_m_ increase of >1 pico-Farad, corresponding to fusion of a membrane equivalent of >10% of IHC plasma membrane and more than 100-fold in excess of the summed RRP of all synapses of an IHC. Hence, it has remained uncertain whether the estimated intrinsic Ca^2+^ dependence of membrane fusion was representative for that of SVs (41). Efforts focusing on RRP exocytosis by whole-cell perforated-patch C_m_ recordings (13) and iGluSNfR imaging of glutamate release at single AZs (14) found smaller *m_Zn_* estimates [C_m_, *m_Zn_*: 3.5 ± 0.1 (SEM) (13); iGluSNfR, *m_Zn_*: 2.5 ± 1.0 (SEM) (14)]. They likely reflect an underestimation of *m* due to partial RRP depletion during the 20 (13) and 10 (14) ms depolarizations, employed for obtaining sufficient signal. By relating the C_m_ increase to the integrated Ca^2+^ influx evoked by 100 ms long depolarizations at different [Ca^2+^]_e_, others concluded on a linear Ca^2+^ dependence of exocytosis (*m*: 0.9) in mouse IHCs after the onset of hearing that was attributed to Ca^2+^ sensing by synaptotagmin IV (71). One caveat of this conclusion is that the findings are impacted by SV replenishment that heavily contributes to exocytosis with prolonged stimulation and is also regulated by Ca^2+^ (11, 12, 15, 72–75) but likely in a different manner than SV fusion. This caveat also affected the most thorough analysis to date of the role of otoferlin as putative Ca^2+^ sensor in IHCs that relied on slow Ca^2+^ uncaging (48). Further testing of the Ca^2+^ sensor hypothesis will ideally employ paired recordings to study the intrinsic Ca^2+^ dependence of SV release from IHCs expressing mutant otoferlin with altered Ca^2+^ binding to one or more C_2_ domains.

Avoiding impact of saturation of exocytosis (i.e., RRP depletion or Ca^2+^ sensor saturation) and of SV replenishment is also relevant when investigating the number of Ca^2+^ channels involved in controlling exocytosis of a given SV. Efforts based on C_m_ recordings and computational modeling indicated that few Ca^2+^ channels [up to 3 of on average 120 channels (34)] couple to a given SV release site with an effective coupling distance of ~15 nm (13, 35, 39) in IHCs of hearing mice. Estimates of *m* were consistently lower when varying Ca^2+^ influx by changes in the number of open Ca^2+^ channels [C_m_, *m_isradipine_*: 1.4 (35) and iGluSNfR, *m_Δ__V_*: 1.5 (14)] than by changes in single channel current (2.5 to 3.5) (13, 32, 33, 35). The present rigorous analysis of initial SV release in response to 5-ms depolarization revealed the strongest *m* difference for these manipulations reported for IHCs to our knowledge: *m_isradipine_*: 1.5 vs. m*_Zn_*: 4.3. In addition, we could estimate release probability while biophysically titrating the number of open Ca^2+^ channels contributing fusogenic Ca^2+^ ions during deactivating Ca^2+^ (tail) currents (61, 66, 67, 76). The *m* estimate by this approach (*m_tail_*: 0.9 to 1.4) was generally consistent with *m_isradipine_* but seems more reliable than the progressive block of Ca_V_1.3 channels by isradipine (e.g., mode of drug action, potential rundown of release). This *m_tail_* is substantially lower than those estimated for the calyx of Held synapse in the auditory brainstem of hearing mice (3–4) (61, 67) indicating tighter coupling of Ca^2+^ channels and SV release sites with less overlap of the Ca^2+^ domains of the triggering channel(s) at the AZs of IHC in hearing mice.

While both synapses are tasked with high rates of temporally precise transmission of auditory information, they differ greatly in structure and molecular composition (77, 78). Hair cells contain high concentrations (mM) of endogenous mobile Ca^2+^ buffers (39, 62, 79), and IHC synapses feature a single AZ with on average 120 Ca^2+^ channels of Ca_V_1.3 (13, 24, 34) and seem to employ otoferlin (46–48), instead of synaptotagmins 1 or 2 (80, 81), as Ca^2+^ sensor of SV fusion. In contrast, the calyx of Held is less heavily buffered e.g. ref. 82, holds hundreds of small AZs (83) with on average 20 to 30 primarily Ca_V_2.1 channels (84, 85), and employs synaptotagmin 2 as Ca^2+^ sensor of release (86). These properties enable the specific and distinct synapse functions: encoding of all sound information driven by the IHC receptor potential and reliable action potential-driven transmission for calculation of interaural time differences in the olivary complex.

Sound encoding requires the stochastically operating IHC AZ with approximately a dozen SV release sites to transmit precise temporal information for sounds of different intensities e.g. ref. 10. Transfer functions of hair cell synapses exhibit large linear portions (12–16, 87) which can be attributed to their Ca^2+^ nanodomain-like coupling (1, 77). In fact, the *m*_Δ__V_** indicates that Ca^2+^ nanodomain-like coupling prevails in the range of receptor potentials. We hypothesize that the supralinear intrinsic Ca^2+^ dependence and low Ca^2+^ affinity binding (effective K_D_ of IHC exocytosis for Ca^2+^: 70 µM) (52) of SV release reflects the properties of the Ca^2+^ sensor of SV release. Employing a multi-C_2_-domain Ca^2+^ sensor such as otoferlin might be seen as a wasteful investment when the sensor operates in the saturating range of the intrinsic Ca^2+^ dependence of exocytosis (52) due to its control by the Ca^2+^ nanodomain [>100 µM (34, 62)]. However, we would like to point out that, this way, the topography of IHC AZs achieves optimal tracking of the receptor potential by synaptic transmission while being least susceptible to influences arising from Ca^2+^ signals of lower amplitude resulting from mechanoelectrical transduction or potential efferent synaptic transmission.

Interestingly, our analysis revealed little variability in the apparent Ca^2+^ dependence for the various protocols among the synapses that we approached from the modiolar side, where a previous imaging study had revealed substantial variability in *m* including high values of *m_Zn_* [up to 8 (14)]. Reasons likely to contribute include differences in pulse duration, spatial and kinetic properties of glutamate detection and its sensitivity, relating release to synaptic vs. IHC Ca^2+^ influx. Clearly, embracing synaptic heterogeneity by the paired recordings will require a larger sample size and ideally reliable tracking of synapse position (14, 88) and/or SGN subtype (89), and rate of spontaneous transmission (88, 89) to relate presynaptic IHC AZ and SGN properties. Moreover, it will be interesting yet challenging to relate the glutamate release to the Ca^2+^ signal at the AZ. This would overcome the caveat of the present analysis of relating changes in SV release at single AZs to the whole-cell Ca^2+^ current that sums over all synaptic and extrasynaptic Ca^2+^ channels (13, 34, 35).

## Materials and Methods

### Animals and Tissue Preparation.

c57BL/6N mice of either sex between postnatal days 14 to 23 (p. 14–23) were used. The animal handling and experiments complied with national animal care guidelines and were approved by the University of Göttingen Board for animal welfare and the Animal Welfare Office of the State of Lower Saxony. Animals were killed by decapitation, and the cochleae were extracted in modified HEPES Hanks’ solution containing 5.36 mM KCl, 141.7 mM NaCl, 1 mM MgCl_2_-6H_2_O, 0.5 mM MgSO_4_-7H_2_O, 10 mM HEPES (4-(2-hydroxyethyl)-1-piperazineethanesulfonic acid), 0.5 mg/mL L-glutamine, and 1 mg/mL D-glucose (pH 7.2, osmolarity of ~300 mOsm). The apical coil of the organ of Corti was dissected and placed under a grid in the recording chamber. Pillar or modiolar supporting cells were removed using soda glass pipettes in order to gain access to the basolateral face of the IHCs and to the postsynaptic boutons of type I SGNs. Dissection of the organ of Corti and cleaning of the supporting cells were performed at room temperature (20 to 25 °C).

### Electrophysiological Recordings.

Pre- and postsynaptic paired patch clamp recordings were performed at near physiological temperature (32 to 37 °C) using an EPC-9 amplifier (HEKA electronics). Patch electrodes were positioned using a PatchStar micromanipulator (Scientifica, UK). Whole-cell recordings from IHCs were achieved using the perforated-patch clamp technique (11) using Sylgard™-coated borosilicate pipettes with an outer diameter of 1.5 mm and typical resistances between 3.5 and 6 MΩ. The IHC pipette solution contained 129 mM Cs-gluconate, 10 mM tetraethylammonium (TEA)-Cl, 10 mM 4-AP, 10 mM HEPES, 1 mM MgCl_2_ (pH 7.2, osmolarity of ~290 mOsm), as well as 300 μg/mL amphotericin B added prior to the experiment. Once the series resistance of the IHC reached below 30 MΩ, whole-cell voltage-clamp recordings from a contacting bouton were performed largely as described in previous studies (17, 90, 91). Sylgard™-coated borosilicate pipettes with an outer diameter of 1.0 mm and typical resistances between 7 and 12 MΩ were used for the postsynaptic recordings. The bouton pipette solution contained: 137 mM KCl, 5 mM ethylene glycol-bis(β-aminoethyl ether)-N,N,N',N'- tetraacetic acid (EGTA), 5 mM HEPES, 1 mM Na_2_-guanosine triphosphate (Na2-GTP), 2.5 mM Na_2_-Adenosine triphosphate (Na2-ATP), 3.5 mM MgCl_2_·6H_2_O and 0.1 mM CaCl_2_ (pH 7.2 and osmolarity of ~290 mOsm). For most recordings, the organ of Corti was continuously perfused with an extracellular solution containing 4.2 mM KCl, 95 to 100 mM NaCl, 25 mM NaHCO_3_, 30 mM TEA-Cl, 1 mM Na-Pyruvate, 0.7 mM NH_2_PO_4_·H_2_O, 1 mM CsCl, 1 mM MgCl_2_·H_2_O, 1.3 mM CaCl_2_, and 11.1 mM D-glucose (pH 7.3, osmolarity of ~310 mOsm). Then, 2.5 µM tetrodotoxin (Tocris or Santa Cruz) was added to block voltage-gated Na^+^ channels in the postsynaptic bouton. The extracellular solution was continuously aerated with carbogen (95% O2, 5% CO2). For 8 pairs included in Fig. 5 (*SI Appendix*, Fig. S5 *A*, *i–viii*) and three pairs in Fig. 4 (*SI Appendix*, Fig. S5 *A*, *i–iii*), the extracellular solution contained 2.8 mM KCl, 110 mM NaCl, 10 mM HEPES, 35 mM TEA-Cl, 2 mM Na-Pyruvate, 0.7 mM NH_2_PO_4_·H_2_O, 1mM CsCl, 0.9 mM MgCl_2_·H_2_O, 1.3 mM CaCl_2_ (except stated otherwise in the figure), and 11.1 mM D-glucose (pH 7.3, osmolarity of ~310 mOsm).

Data were acquired using the Patchmaster software (HEKA electronics). The current signal was sampled at 20 to 50 kHz and filtered at 5 to 10 kHz. IHC were voltage-clamped at a holding potential of −58 mV, corresponding to the presumable in vivo resting potential (53). Two IHCs were voltage-clamped at −69 mV. The boutons were held at a potential of −94 mV. All reported potentials are corrected by the liquid junction potential (19 mV for the IHC and 4 mV for the bouton). Ca^2+^ current recordings were corrected for linear leak current using a *P/n* protocol. We excluded IHCs and boutons with leak currents exceeding −60 pA and −100 pA at holding potential, respectively (with the exception of one pair with bouton leak around −800 pA). The series resistance of the IHCs was typically below 30 MΩ. The apparent series resistance of the bouton was calculated from the capacitive transient in response to a 10 mV test pulse. The actual series resistance (R_s_) was calculated offline as previously reported (91). The bouton Rs was typically below 80 MΩ.

The apparent Ca^2+^ dependence of neurotransmitter release was studied using 2 to 5 ms step-depolarizations and using different intensities of depolarization or the slow perfusion of Ca^2+^ channel blockers to vary the Ca^2+^ influx into the IHC. For the latter, isradipine (Sigma-Aldrich) or ZnCl_2_ (Sigma-Aldrich) were added to the extracellular solution and slowly perfused into the chamber while recording the responses to 5 ms depolarization pulses. Isradipine was diluted to a final concentration of 0.5 to 2 µM from a stock of 20 mM in DMSO. ZnCl_2_ was diluted to a final concentration of 1 mM from a stock of 0.1 M and filtered with a pore size of 0.2 µm. The time interval between two subsequent depolarizations was at least 10 s. In addition, we used a tail current protocol to study release in response to graded numbers of open Ca^2+^ channels. The time interval between two subsequent tail protocols was 3.5 s. The recording time for each pair is reported in Table 1.

**Table 1.** Total time of the recording for each pair included in Figs. 2–5

Pair #	Total recording time (s)	Manipulation
l300817_2	968	Zn
l161117_1	822	Zn
l100118_2	565	Zn
l120118_1	964	Zn
l230118_1	736	Zn
l280218_1	988	Zn
l080118_1	1,564	Zn
l040917_1	392	Isradipine
l050917_1	1,262	Isradipine
l050917_2	1,278	Isradipine
l010318_2	892	Isradipine
l220318_1	936	Isradipine
l100418_1	2,717	Isradipine
l050318_1	3,637	Isradipine
l230817_2	452	Tails
l280817_1	673	Tails
l161017_1	117	Tails
l071016_1	352	Tails
l020916_1	265	Tails
l090916_1	763	Tails
l200418_1	290	Tails
l081020_1	410	Tails
l071016_1	240	ΔV
l241116_1	372	ΔV
l020117_1	358	ΔV
l030117_1	384	ΔV
l050117_1	665	ΔV
l050517_1	737	ΔV
l140817_1	707	ΔV
l140817_2	544	ΔV
l140817_3	639	ΔV
l300817_1	857	ΔV
l240518_1	1,579	ΔV
l180618_1	1,677	ΔV
l240117_1	480	ΔV
l240217_4	378	ΔV

### Data Analysis.

Electrophysiological data were analyzed using the IgorPro 6 Software Package (Wavemetrics), GraphPad Prism 10, and Excel. Ca^2+^ (Q_Ca_) and EPSC charge (Q_EPSC_) were estimated by taking the integrals of the currents.

Ca^2+^-dependence of release for individual pairs was determined by fitting the Q_EPSC_ vs. IHC Q_Ca_ plots with a power function:

$$Q_{\text{EPSC}}=a{\left(Q_{\text{Ca}}\right)}^{m},$$

where *m* corresponds to the Ca^2+^ cooperativity. Some pairs showed a clear saturation of release at high IHC Q_Ca_. In these cases, the fit was restricted to the datapoints before the plateau, which was determined by visual inspection and a sigmoid fit. For the pooled data, the power function was fitted to the normalized Q_EPSC_ vs normalized Q_Ca_. For the pairs with saturation of release, Q_Ca_ was normalized to a point before the plateau.

Data were prepared for presentation using Adobe Illustrator. Statistical significance was assessed in GraphPad Prism 10 using a Brown–Forsythe and Welch ANOVA tests followed by a Dunnett’s T3 multiple comparisons test. Data are expressed as mean ± SEM and SD.

## Supplementary Material

Appendix 01 (PDF)

Appendix 02 (PDF)

## Data Availability

Original data created for the study are available at the Research Data Repository of the Göttingen Campus (GRO.data) with the DOI/accession number https://doi.org/10.25625/MUZZJN (92).
