# Supplementary material for: Ca2+ regulation of glutamate release from inner hair cells of hearing mice
Source: Proc Natl Acad Sci U S A. 2023 Nov 29;120(49):e2311539120. doi: 10.1073/pnas.2311539120 (PMC10710057; doi:10.1073/pnas.2311539120)
Supplement: Supplementary file 2 — Appendix 02 (PDF) [file pnas.2311539120.sapp2.pdf]

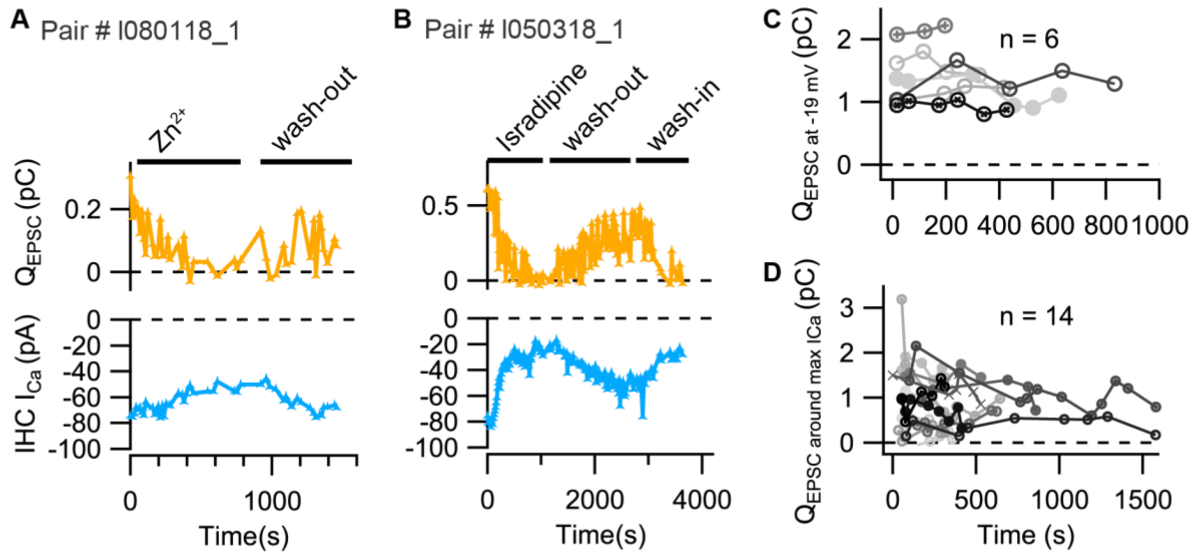

Figure S1 Testing for the potential rundown of exocytosis during manipulations of Ca^{2+} influx

A,B. Related to Fig. 2 and Fig.3: slow perfusion of Zn^{2+} (A) or isradipine (B) progressively reduces the whole-IHC Ca^{2+} current (I_{Ca}; blue) and the concomitant evoked EPSC charge (Q_{EPSC}; orange). Slow wash-out using normal extracellular solution partially restores I_{Ca} and neurotransmitter release for both pharmacological manipulations. In B, wash-in of isradipine once again reduces I_{Ca} and Q_{EPSC}. **C.** Related to Fig. 4: during tail-current experiments, rundown of exocytosis was probed as changes in Q_{EPSC} elicited by 2 or 10 ms voltage steps to -19 mV over time. We recorded these periodic depolarizations after each full set of tail current protocols in 6 pairs. **D.** Related to Fig. 5: to address potential rundown of exocytosis, Q_{EPSC} elicited by 2 ms pulses at voltages eliciting maximal Ca^{2+} influx (-19, -21 and -23 mV) was plotted vs. time (n = 14 pairs).

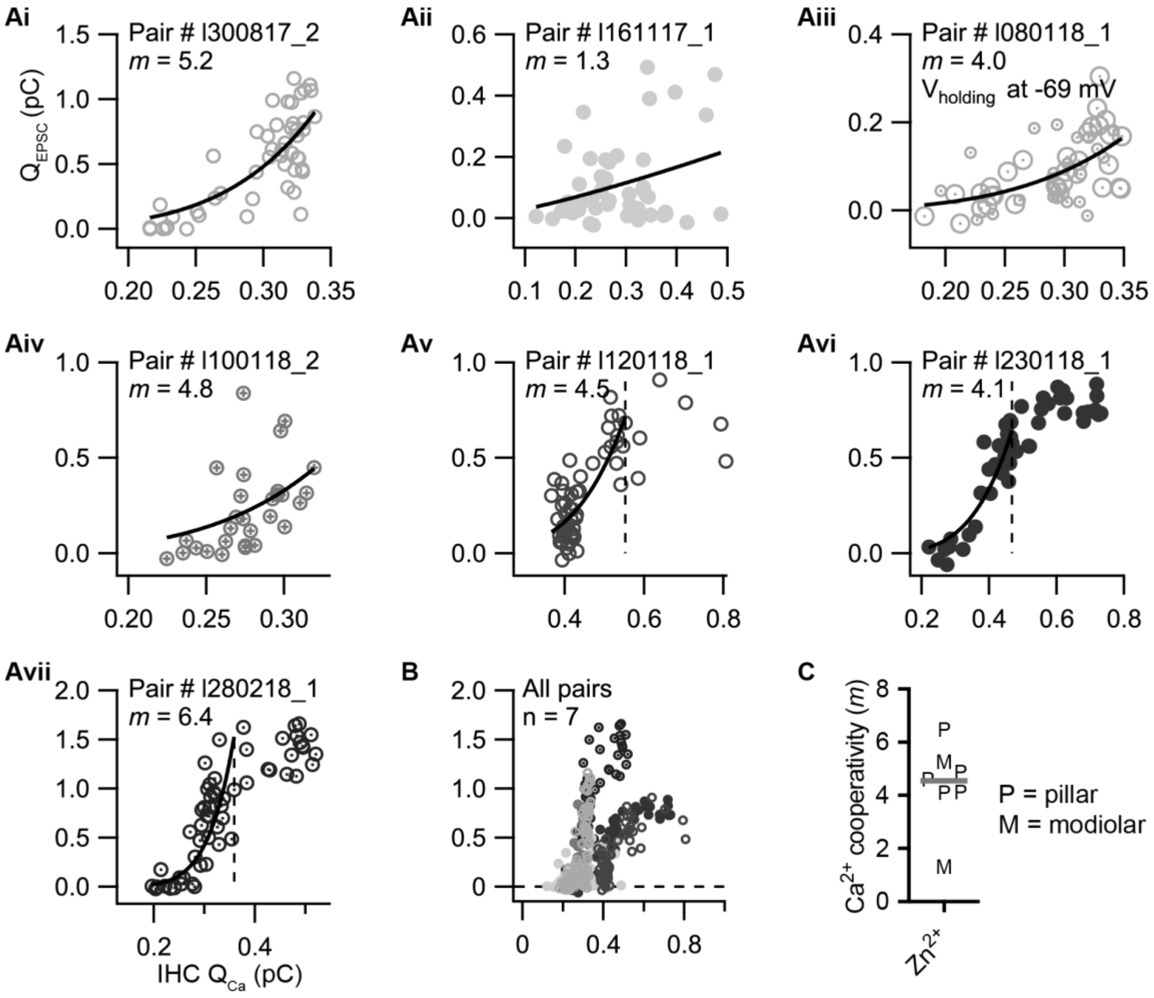

Figure S2 Estimating the intrinsic Ca^{2+} dependence of SV release for each individual pair

Ai-Avii. Scatter plots of the EPSC charges (Q_{EPSC}) vs. the corresponding Ca^{2+} current integrals (Q_{Ca}) for each individual pair during perfusion of 1 mM Zn^{2+} to reduce the *effective* fusogenic Ca^{2+}. The solid line is a least-squares fit of a power function ($Q_{EPSC} = a(Q_{Ca})^m$) to each pair data. For pair # I080118_1 (**Aiii**), the smaller markers represent the data recorded while washing-out Zn^{2+}, which was also included in the fitting. **B.** Scatter plot of the EPSC charges (Q_{EPSC}) vs. the corresponding Ca^{2+} current integrals (Q_{Ca}) of all pairs: different markers and shades of gray for the different pairs (n = 7). **C.** Ca^{2+} cooperativity (*m*) estimated for each individual pair shown in A. P indicates the boutons contacting the pillar side of the IHC; M indicates the boutons contacting the modiolar side of the IHC. Gray bar corresponds to the median.

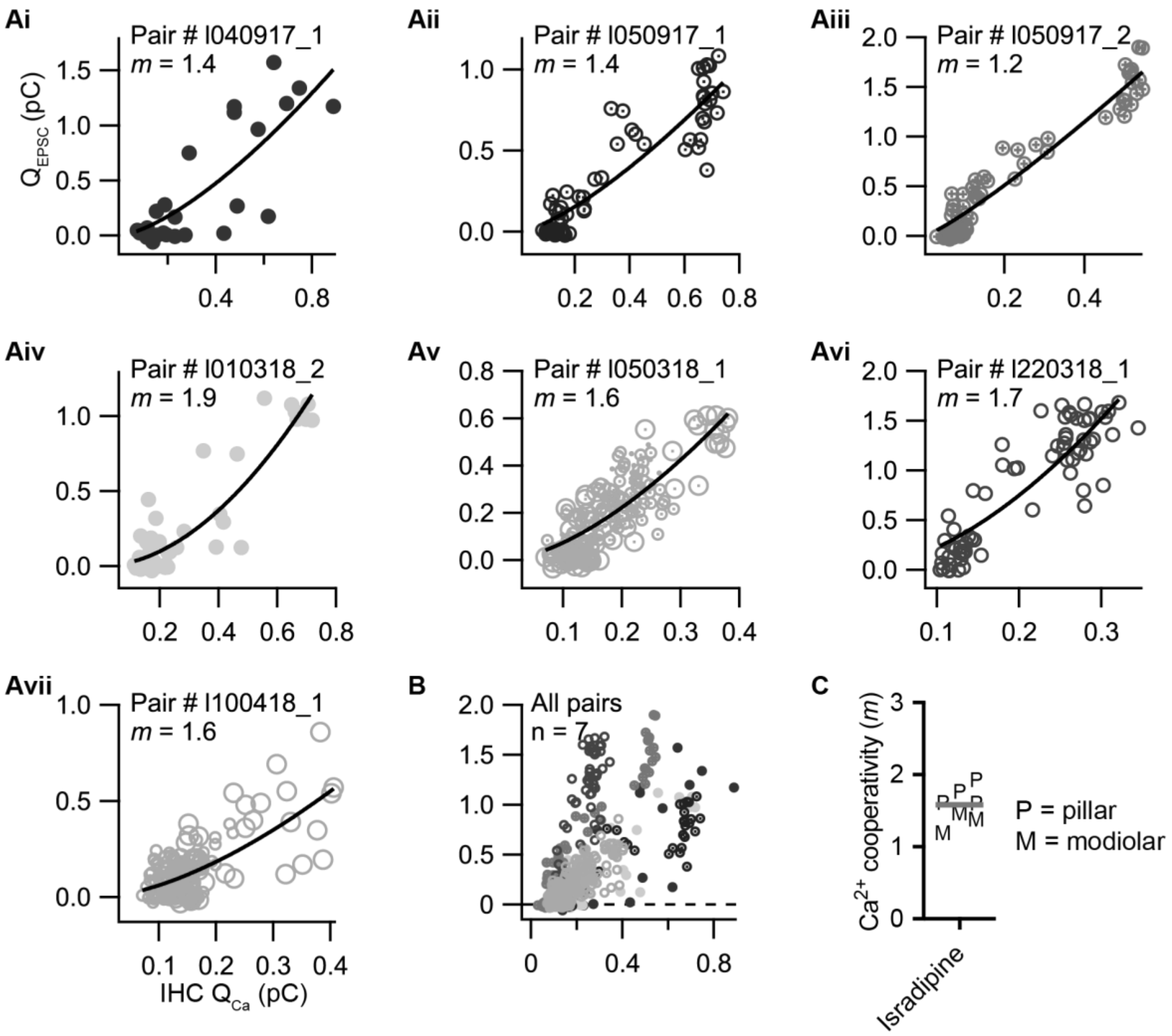

Figure S3. Apparent Ca^{2+} dependence of SV release for each individual pair during isradipine application

Ai-Avii. Scatter plots of the EPSC charges (Q_{EPSC}) vs. the corresponding Ca^{2+} current integrals (Q_{Ca}) for each individual pair during perfusion 0.5 – 2 µM of the dihydropyridine isradipine to progressively shift the Ca^{2+} channels to a non-conducting state. The solid line is a least-squares fit of a power function ($Q_{EPSC} = a(Q_{Ca})^m$) to each pair data. For pairs # I050318_1 and # I100418_1, the smaller markers represent the data recorded while washing-out isradipine, which was also included in the fitting. **B.** Scatter plot of the EPSC charges (Q_{EPSC}) vs. the corresponding Ca^{2+} current integrals (Q_{Ca}) of all pairs: different markers and shades of gray for the different pairs (n = 7). **C.** Ca^{2+} cooperativity (m) estimated for each individual pair shown in A. P indicates the boutons contacting the pillar side of the IHC; M indicates the boutons contacting the modiolar side of the IHC. Gray bar corresponds to the median.

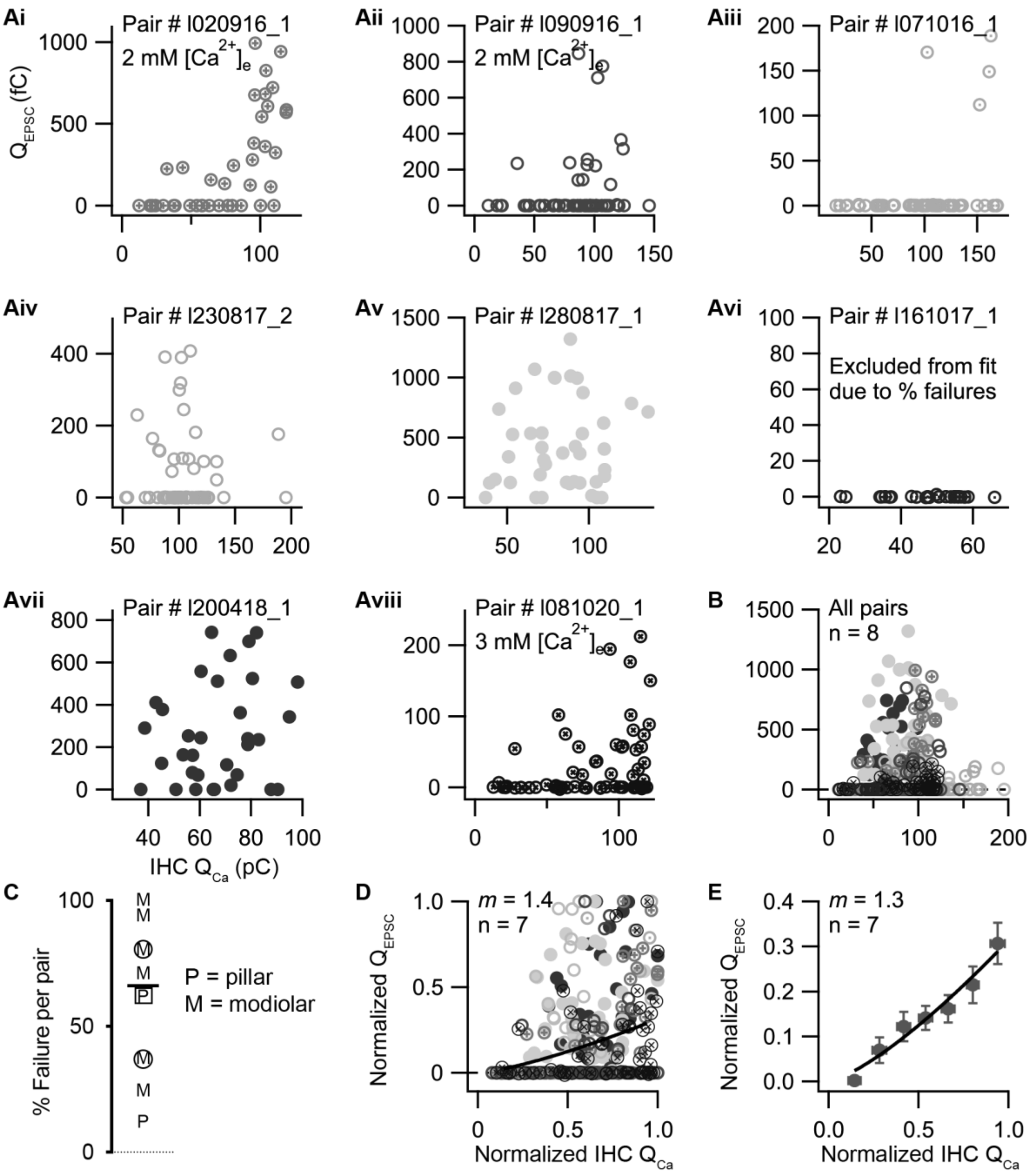

Figure S4. Apparent Ca^{2+} dependence of SV release during Ca^{2+} tail-current experiments

Ai-Aviii. Scatter plots of the EPSC charges (Q_{EPSC}) vs. the corresponding Ca^{2+} current integrals (Q_{Ca}) for each individual pair during deactivating (tail) currents upon repolarization **B.** Scatter plots of the EPSC charges (Q_{EPSC}) vs. the corresponding Ca^{2+} current integrals (Q_{Ca}) for all pairs: different markers and shades of gray for the different pairs (n = 8). **C.** Percentage of failure of synaptic transmission for each individual pair shown in A. P indicates the boutons contacting the pillar side of the IHC; M indicates the boutons contacting the modiolar side of the IHC. Circles and squares are the pairs recorded at 2 mM and 3 mM extracellular [Ca^{2+}], respectively. Black bar represents to the median. **D.** Scatter plots of the normalized EPSC charges (Q_{EPSC}) vs. the corresponding normalized Ca^{2+} current integrals (Q_{Ca}) for all pairs: different markers and shades of gray for the different pairs (n = 7). The solid line is a least-squares fit of a power function ($Q_{EPSC} = a(Q_{Ca})^m$) to the normalized population data for Q_{EPSC} and Q_{Ca} yielded m_{tails} of 1.4 (n = 7 pairs). **E.** Power function fit to the binned normalized data (bin size ~ 0.15;

data points are mean ± SEM) from (C) resulted in m_{tails} of 1.3.

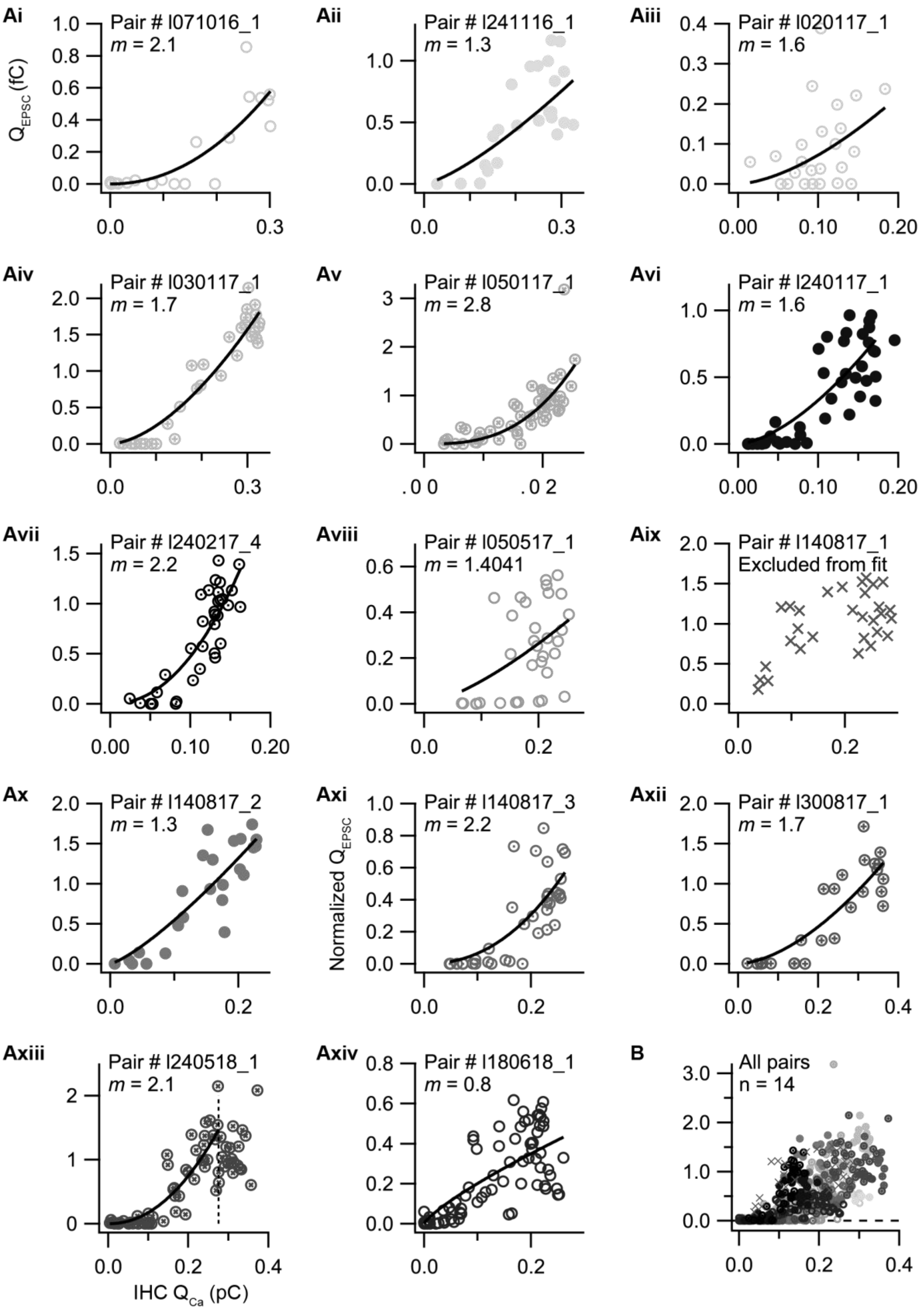

Figure S5. Apparent Ca^{2+} dependence of SV release in the range of IHC receptor potentials

Ai-Axiv. Scatter plots of the EPSC charges (Q_{EPSC}) vs. the corresponding Ca^{2+} current integrals (Q_{Ca}) for each individual pair in response to 2 ms depolarizations to randomized voltages in the hyperpolarized range. The solid line is a least-squares fit of a power function ($Q_{EPSC} = a(Q_{Ca})^m$) to each pair data. Pair #

l140817_1 (Aix) was excluded because release was not completely abolished even at resting potential. **B.** Scatter plots of the EPSC charges (Q_{EPSC}) vs. the corresponding Ca^{2+} current integrals (Q_{Ca}) for all pairs: different markers and shades of gray for the different pairs (n = 14).
